# Sulfamoylated Estradiol Analogs Targeting the Actin and Microtubule Cytoskeletons Demonstrate Anti-Cancer Properties In Vitro and In Ovo

**DOI:** 10.3390/cancers16172941

**Published:** 2024-08-23

**Authors:** Anne Elisabeth Mercier, Anna Margaretha Joubert, Renaud Prudent, Jean Viallet, Agnes Desroches-Castan, Leanne De Koning, Peace Mabeta, Jolene Helena, Michael Sean Pepper, Laurence Lafanechère

**Affiliations:** 1 Department of Physiology, School of Medicine, Faculty of Health Sciences, University of Pretoria, Pretoria 0028, South Africa; annie.joubert@up.ac.za (A.M.J.); peace.mabeta@up.ac.za (P.M.); jolene.giliam@fortrea.com (J.H.); laurence.lafanechere@univ-grenoble-alpes.fr (L.L.); 2 Institute for Advanced Biosciences, INSERM U1209, CNRS UMR5309, Université Grenoble Alpes, 38000 Grenoble, France; renaud_prudent@yahoo.fr; 3 Inovotion SAS France, Biopolis, 38700 La Tronche, France; jean.viallet@inovotion.com; 4 Laboratoire Biosanté U1292, Université Grenoble Alpes, Inserm, CEA, 38000 Grenoble, France; agnes.castan@inserm.fr; 5 Institut Curie Centre de Recherche, PSL Research University, 75248 Paris Cedex 05, France; leanne.de-koning@curie.fr; 6 Institute for Cellular and Molecular Medicine, Department of Immunology, and South African Medical Research Council Extramural Unit for Stem Cell Research and Therapy, School of Medicine, Faculty of Health Sciences, University of Pretoria, Pretoria 0028, South Africa; michael.pepper@up.ac.za; 7 Team Cytoskeleton Dynamics and Nuclear Functions, Institute for Advanced Biosciences, INSERM U1209, CNRS UMR5309, Université Grenoble Alpes, 38000 Grenoble, France

**Keywords:** microtubules, actin, anti-angiogenic, metastasis, cofilin, ezrin/radixin/moesin, chick chorioallantoic membrane assay (CAM), migration, invasion, cancer, 2-methoxyestradiol analogs

## Abstract

**Simple Summary:**

The naturally occurring derivative of estrogen, 2-methoxyestradiol (2-ME), has been shown to have good anti-cancer properties. However, it is broken down too quickly within the blood to be clinically useful. We designed 2-ME analogs with modifications which could avoid rapid metabolism, would preferably stay in the tumor, and were more toxic to cancer cells. Here, we looked more closely at how these compounds work within cancer cells, and how they communicate within themselves and with their environment. ESE-15-one and ESE-16 interfere with the functioning of the intracellular cytoskeleton (actin and microtubules), sending messages that stop cell division, intracellular transport of proteins, and cell migration and invasion, eventually inducing cell suicide. They also inhibited the movement of cells that make blood vessels that would support tumor growth. Using eggs with chicken embryos, we could show that the compounds reduced the tumor size and diminished the spread of cancer cells in a living system.

**Abstract:**

The microtubule-disrupting agent 2-methoxyestradiol (2-ME) displays anti-tumor and anti-angiogenic properties, but its clinical development is halted due to poor pharmacokinetics. We therefore designed two 2-ME analogs in silico—an ESE-15-one and an ESE-16 one—with improved pharmacological properties. We investigated the effects of these compounds on the cytoskeleton in vitro, and their anti-angiogenic and anti-metastatic properties in ovo. Time-lapse fluorescent microscopy revealed that sub-lethal doses of the compounds disrupted microtubule dynamics. Phalloidin fluorescent staining of treated cervical (HeLa), metastatic breast (MDA-MB-231) cancer, and human umbilical vein endothelial cells (HUVECs) displayed thickened, stabilized actin stress fibers after 2 h, which rearranged into a peripheral radial pattern by 24 h. Cofilin phosphorylation and phosphorylated ezrin/radixin/moesin complexes appeared to regulate this actin response. These signaling pathways overlap with anti-angiogenic, extra-cellular communication and adhesion pathways. Sub-lethal concentrations of the compounds retarded both cellular migration and invasion. Anti-angiogenic and extra-cellular matrix signaling was evident with TIMP2 and P-VEGF receptor-2 upregulation. ESE-15-one and ESE-16 exhibited anti-tumor and anti-metastatic properties in vivo, using the chick chorioallantoic membrane assay. In conclusion, the sulfamoylated 2-ME analogs displayed promising anti-tumor, anti-metastatic, and anti-angiogenic properties. Future studies will assess the compounds for myeloproliferative effects, as seen in clinical applications of other drugs in this class.

## 1. Introduction

Microtubule-disrupting agents (MDAs) are widely used in cancer therapy with proven efficacy [1]. Although the introduction of these drugs has changed the clinical course of cancer prognostics, the emergence of multidrug resistance and adverse side effects are prompting the development of MDAs with improved properties. The endogenous breakdown product of 17β-estradiol, (17 beta)-2-methoxyestra-1,3,5(10)-triene-3,17-diol, or 2-methoxyestradiol (2-ME), initially seemed to be a promising anti-cancer compound. Pre-clinical studies reported effective cytotoxicity of 2-ME in numerous cell lines in vitro, and anti-tumor activity in vivo [2,3,4]. Reports of the preferential sparing of non-neoplastic cells, the evasion of P-glycoprotein efflux pumps, the absence of myelosuppression, as well as antiangiogenic properties, encouraged progression to phase II clinical trials, in which 2-ME was registered as Panzem^®^ [5,6].

**Figure 1:**
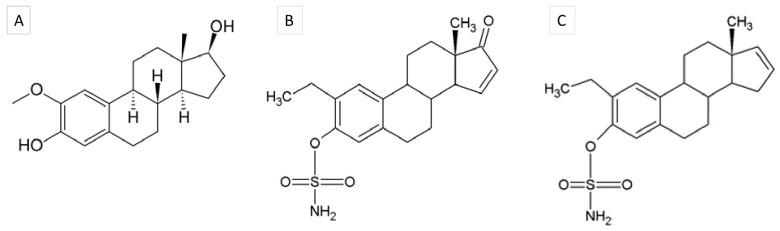
Chemical structures of (**A**) (17 beta)-2-methoxyestra-1,3,5(10)-triene-3,17-diol (2-methoxyestradiol), (**B**) ESE-15-one (2-ethyl-3-O-sulphamoyl-estra-1,3,5(10),15-tetraene-3-ol-17-one), and (**C**) ESE-16 (2-ethyl-3-O-sulphamoyl-estra-1,3,5(10)16-tetraene).

However, poor pharmacokinetics halted the clinical development of this drug and prompted the design and development of 2-ME analogs to circumvent this failure [7]. To this end, our team designed a range of sulfamoylated 2-ME analogs in silico by modifying the C2 and C17 positions with moieties known to improve the drug’s anti-mitotic activity and increase its half-life [8]. The synthesis and in-depth study of the anti-cancer properties of two of these compounds, namely 2-ethyl-3-O-sulfamoyl-estra-1,3,5(10),15-tetraene-3-ol-17-one (ESE-15-one) and 2-ethyl-3-O-sulphamoyl-estra-1,3,5(10)16-tetraene (ESE-16) (Figure 1), confirmed their enhanced cytotoxic activity on different neoplastic cell types, including multi-drug-resistant cell lines, with the induction of autophagic and apoptotic cell death [9,10]. As with 2-ME, these analogs induce both a metaphase and a G_1_/S block of the cell cycle, correlated with an increase in p27^Kip1^ and p15^INK4B^expression [11]. In addition, these compounds appear to spare non-neoplastic cells and radiosensitize cancer cells [12,13,14]. We have recently shown that ESE-15-one and ESE-16 do, indeed, behave as MDAs that bind to the colchicine binding site of tubulin and reversibly depolymerize cell microtubules [11].

The present study aimed to kinetically characterize the cellular effects of these compounds on the actin and microtubule cytoskeleton, and to investigate the consequences of their administration on cellular functions involved in the establishment of metastasis, such as cell migration and invasion. Using time-lapse microscopy, we analyzed the effect of these compounds on cell microtubule dynamics. We also investigated their effect on actin filaments at different time points. Then, using reverse phase protein array technology (RPPA) we kinetically analyzed the signaling pathways modulated by these agents. Moreover, several molecules targeting the colchicine site of tubulin, such as combretastatin [15], show a vascular-disrupting agent-type activity, disrupting the blood vessels that perfuse tumors. Because ESE-15-one and ESE-16 also bind to the colchicine site, we examined their possible vascular disrupting activity. Finally, using chick chorioallantoic xenograft tumor assays, we analyzed the anti-tumoral and anti-metastatic activity of these compounds in vivo.

## 2. Materials and Methods

### 2.1. Cell Lines, Culture Methods, and Chemicals

MDA-MB-231 breast cancer cells, human cervical adenocarcinoma (HeLa) cells (passage < 18), and human umbilical vein endothelial cells (HUVECs) (passage 5) were purchased from and verified by the American Tissue Culture Collection (Manassas, VA, USA). Cancer cells were cultured in Dulbecco’s modified Eagle medium (DMEM) (Gibco^®^, Thermo Fisher Scientific, Waltham, MA, USA) with fetal bovine serum (FBS) (10%) (HyClone, Logan, UT, USA), 100 units/mL penicillin, and 100 µg/mL streptomycin (Sigma-Aldrich, St. Louis, MO, USA). HUVECs were cultured in microvascular endothelial cell growth medium 2 (EGM-2-MV) (Cambrex, East Rutherford, NJ, USA) with 5% FBS. Cells were cultured in a humidified environment in a Forma Scientific water-jacketed incubator (37 °C, 5% carbon dioxide). All other reagents were of analytical grade and, if not specifically mentioned, were purchased from Sigma-Aldrich (St. Louis, MO, USA). Buffer constituents are supplied in the Supplementary Methods S1.1.

Ithemba Pharmaceuticals (PTY) Ltd. (Johannesburg Gauteng, South Africa) synthesized the in silico-designed compounds, namely 2-ethyl-3-O-sulphamoyl-estra-1,3,5(10)15-tetraene-3-ol-17-one (ESE-15-one) and 2-ethyl-3-O-sulphamoyl-estra-1,3,5(10)16-tetraene (ESE-16), which were dissolved in dimethyl sulfoxide (DMSO). Cells were exposed to the 0.186 µM ESE-15-one and 0.5 µM ESE-16 (IG_50_ concentrations), with ½ and ¼ of those concentrations used to assess non-lethal effects [9]. Unless otherwise stated, cells (1 × 10^6^) were seeded in 25 cm^2^ flasks, allowing for a 24-hour (h) attachment period, and then exposed to the compounds in parallel to the appropriate controls (DMSO vehicle control and positive experimental controls). On termination, cells were trypsinized and washed with phosphate-buffered saline (PBS).

### 2.2. Microtubule Dynamic Instability Parameters: Time-Lapse Fluorescence Microscopy

End-binding protein 3-tagged with green fluorescent protein (EB3-GFP) plasmids were used to label the microtubule plus ends in a protocol previously described by Honore and Braguer [16]. HeLa cells were transfected with EB3-GFP plasmids via electroporation using the Amaxa^®^ Cell Line Nucleofector^®^ Kit R transfection system for HeLa cells (Amaxa^®^, Koln, Germany). HeLa cells (1 × 10^6^) were re-suspended in Nucleofector^®^ Solution (200 µL). For each 100 µL of cell suspension, 2 µg pmaxGFP^®^Vecor was added. Cells (8 × 10^5^) were transferred per certified cuvette for transfection with the Nucleofector^®^ Program I-013 on the Nucleafector^®^I device (Amaxa^®^, Koln, Germany). Transfected cells (2 × 10^5^) were transferred to Lab-Tek^®^ chambers (Lab-Tek^®^ Chamber #1.0 Borosilicate Coverglass System, Thermo Fisher Scientific, Waltham, MA, USA) using the supplied pipettes (37 °C/5% CO_2_ overnight). ESE-15-one and ESE-16 at various concentrations (0.186 µM, 0.093 µM and 0.04 µM for ESE-15-one and 0.5 µM, 0.25 µM and 0.125 µM for ESE-16) were added for 2 h (37 °C). Colchicine (0.05 μM) was used as a positive control method. Time-lapse fluorescent video-microscopy of the live cells was done using an inverted fluorescent microscope (ZEISS Axiovert 200M with 63× objective). An image was captured every 4 seconds (s) for 7 minutes (min) using CoolSNAP HQ (Roper Scientific, Mercerville, NJ, USA), driven by MetaMorph Version 7.10 software (Universal Imaging Corp, Downingtown, PA, USA). Analysis of images was done using ImageJ Version 1.50i software (NIH, Bethesda, MD, USA) where a minimum of 8 microtubules plus ends were tracked per cell, and a minimum of 6 cells per experimental condition were analyzed. Data from the tracking were analyzed as % time spent growing, % time in pause, growth rate, and catastrophe frequency. Statistical significance was calculated using the Student’s *t*-test.

### 2.3. Fluorescence Microscopy: Effect of the Molecules on the Actin Skeleton

Cells (6 × 10^2^) were seeded on 12 mm round coverslips in 24 well plates and allowed to attach for 48 h before exposure to the molecules for either 2 or 24 h. Cells were fixed in PBS/formaldehyde 4% at 37 °C for 30 min before permeabilization with PBS/Triton^TM^ X-100 0.2% (room temperature (RT) for 15 min). Cells were incubated in Alexa Fluor 488 Phalloidin (Life Technologies, Thermo Fisher Scientific, Waltham, MA, USA) at 1:100 and Hoechst at 1:1000, diluted in PBS-Tween^®^ 20 (PBS-T) 0.1%/BSA 0.3% for 20 min before being rinsed with PBS-T 0.1%. Images of the mounted specimens, viewed with an X63 oil objective on a Zeiss Axio Imager Z1 microscope, were captured by Axiovision Version 4.8 software (Carl Zeiss, Oberkochen, Germany) with an Ocra R2 N/B camera (Hamamatsu Photonics, Hamamatsu, Japan).

### 2.4. Fluorescence Microscopy: Effect of the Molecules on the Microtubules

HUVECs were grown for 48 h on glass coverslips placed in the wells of 24-well plates (30,000 cells/well). Cells were incubated for 24 h with or without compounds, as indicated in the figure legends. Colchicine (0.1 µM) (Sigma-Aldrich, MO, USA) served as a positive control for cytoskeletal disruption. After treatment, the culture medium was removed and cells were fixed for 20 min at RT with 2% paraformaldehyde in PBS and then permeabilized with 0.2% Triton X-100 in PBS for 5 min. Cells were processed for immunofluorescence after a 1-h saturation step using 2% bovine serum albumin (BSA) in 0.2% PBS-T, as described in Paturle-Lafanechere et al., 1994 [17] using 1:4000 primary rat anti-tyrosinated tubulin (YL1/2) and 1:4000 primary rabbit anti-detyrosinated tubulin (L4) antibodies (Vassal et al., 2006) [18]. Fluorescence images were taken using a Zeiss laser scanning microscope 880 with Airyscan (Jena, Germany) (Supplementary Methods S1.2).

### 2.5. Reverse Phase Protein Array Analyses

Cells were exposed to 0.186 µM ESE-15-one and 0.5 µM ESE-16 for 30 min, 1 h, 2 h, 6 h, 12 h, 18 h, and 24 h. Cells were rinsed with PBS and scraped from the plate in hot Laemmli buffer. After centrifugation (15,000 rpm for 10 min) and incubation (10 min at 100 °C), the supernatants were passed through a fine needle multiple times to reduce the viscosity. The protein concentration of the samples was determined using the Pierce™ BCA protein assay kit (Thermo Fisher Scientific, Waltham, MA, USA). Each sample (three biological repeats, each with *n* = 2) was spotted onto Grace Bio-Labs ONCYTE^®^ SuperNOVA^™^ slides covered in nitrocellulose (Grace Biolabs, Bend, OR, USA) in 5 serial dilutions (2000–125 µg/mL) using a 2470 arrayer (Aushon Biosystems, Billerica, MA, USA). An Autostainer Plus (Dako, Agilent Technologies, Santa Clara, CA, USA) was used to label the arrays with antibodies previously validated for their specificity (Table S1 in the Supplementary Methods). Arrays were saturated in Tris-buffered saline with 0.1% Tween 20 and 5% BSA (TBS-T-BSA) after they had been incubated in biotin, avidin, and peroxidase blocking reagents (Dako, Agilent Technologies, Santa Clara, CA, USA). Arrays were incubated with primary antibodies diluted in TBS-T-BSA (4 °C, 8 h) and washed with TBS-T before incubation (1 h, RT) with the appropriate secondary antibodies coupled to horseradish peroxidase (Jackson ImmunoResearch Laboratories, Newmarket, UK). Slides were probed with Alexa647-Streptavidin (Thermo Fisher Scientific, Waltham, MA, USA) in TBS-T-BSA (1 h at RT), after incubation in Bio-Rad amplification reagent (Bio-Rad, Watford, UK) (15 min, RT) and a TBS-T wash step. Total protein staining was achieved with incubation in 7% acetic acid and 10% methanol (15 min) flowed by a 10 min incubation in Sypro Ruby (Thermo Fisher Scientific, Waltham, MA, USA). Arrays were scanned with the GenePix 4000B microarray scanner (Molecular Devices, San Jose, CA, USA) and microVigene software Version 3.0.0.31587 (VigeneTech Inc., Carlisle, MA, USA) was used to determine the intensity of the spots. The data were normalized using Normacurve [19], with each slide scaled and centered around the median value. The data from each array were corrected for loading effects by applying linear regression to remove dependencies on the median value across all 84 arrays.

### 2.6. Western Blots

After a 24-h attachment (6.0 × 10^5^ cells/well in 6-well plates), cells were exposed to the compounds for 0, 0.5, 1, 2, 6, and 24 h before being washed with ice-cold TBS. Cells were lysed with ice-cold RIPA buffer, collected by scraping, and centrifuged at 200,000 rpm at 4 °C for 30 min in an Optima MAX-XP Beckman ultracentrifuge (Beckman Coulter, Brea, CA, USA) to collect the supernatant. The protein concentration of each sample was determined using the Pierce^®^ BCA protein assay kit (Thermo Fisher Scientific, Waltham, MA, USA), per manufacturer protocol. Denatured (96 °C, 5 min) cellular protein (20 µg) in bromophenol blue (Sigma-Aldrich, St. Louis, MO, USA) was separated via electrophoresis with the Mini-rotean 3 system (Bio-Rad Laboratories Inc., Hercules CA, USA) in 1× migration buffer for 1 h at 0.03 amps on 10% sodium dodecyl sulphate polyacrylamide gels (hand-cast). Polyvinylidene difluoride membranes (Immobilon-P IPVH00010, Merck Millipore Corp., Darmstadt, Germany) were activated in 100% ethanol before the protein transfer using the Mini-Protean 3 system (Bio-Rad Laboratories Inc., Hercules CA, USA) (100 V for 75 min in transfer buffer). Membranes were blocked with TBS-T-BSA for 1 h at RT after being washed in TBS (pH 7.4). Membranes were incubated with cofilin P-S3 raised in rabbit or anti-cofilin raised in rabbit, both from Cell Signaling Technology Corp. (Danvers, MA, USA) (1:1000 antibody in TBS-T-BSA, 4 °C, 8 h). Membranes were washed four times with TBS-T and incubated with horseradish peroxidase-conjugated (HRP) anti-rabbit from Jackson ImmunoResearch (West Grove, PA, USA) at 1:10,000 in TBS-T-BSA. Protein bands were visualized using the ECL prime chemiluminescence kit on Amersham Hyperfilm ECL with an automated X-ray hyperprocessor (all from Amersham Biosciences, GE Healthcare, Chicago, IL, USA). Proto-oncogene tyrosine-protein kinas sarcoma (Src) raised in rabbit (Cell Signaling Technology Corp., Danvers, MA, USA) or mouse monoclonal IgG_2α_ heat shock protein 90 (HSP90 F-8) (Santa Cruz Biotechnology, Dallas, TX, USA), previously shown to have no change in expression [11], were used as loading controls. Secondary antibodies included anti-rabbit HRP (1:10,000 in TBS-T-BSA) from Jackson ImmunoResearch (West Grove, PA, USA) or anti-mouse HRP (1:3000 in TBS-T-BSA) acquired from Sigma-Aldrich, St. Louis, MO, USA. Semi-quantitative densitometric analysis of the bands was performed using Image-J software (NIH, Bethesda, MD, USA), with adjustments made relative to the loading controls. Results were expressed as a fold-decrease or -increase compared to the DMSO vehicle control. The ratio of cofilin expression versus cofilin phosphorylation was calculated for each time point.

### 2.7. Wound Healing Assays

Cells were seeded in a 24-well plate with 1 × 10^5^ cells per well, grown to confluence. After an artificial wound was created with a sterilized 1 mL pipette tip, the wells were rinsed with PBS to remove detached cells, and media containing the required compounds (IG_50_ and ½ IG_50_ concentrations of ESE-15-one and ESE-16) were added. Colchicine (0.5 µM) was used as a positive control. Micrographs were taken at regular time intervals. MDA-MB-231 cell wounds were assessed over 25 h. MDA-MB-231 cells have a doubling time of about 28–38 h [20,21]. Analysis entailed determining the percentage of wound closure compared to the control wound. Nine areas per wound were measured per micrograph. Experiments were conducted in triplicate, n of 3.

### 2.8. Matrigel^TM^ Invasion Assay

A total of 5 × 10^4^ MDA-MB-231 cells were plated on top of a thick layer of Matrigel^TM^ in Transwell chambers (Biocoat, BD Biosciences, Franklin Lakes, NJ, USA) and placed in 10% FCS-supplemented DMEM (in 24-well plates). After 2 h, compounds were added for 24 h (0.093 µM and 0.186 µM for ESE-15-one, and 0.25 µM and 0.5 µM for ESE-16). Selumetinib (5 µM) was used as a positive control [22]. Membranes were examined under a light microscope, and cells which had migrated through the Matrigel^TM^ and reached the bottom of the Transwell membrane were counted. Results were reported as fold-decrease in cells which migrated through the membrane pores when compared to the DMSO vehicle control. Experiments were done in duplicate, n of 3.

### 2.9. Anti-Angiogenic Properties of ESE-15-One and ESE-16: Human Umbilical Vein Endothelial Cell-Migration Test

Confluent HUVEC monolayers were wounded with a sterile plastic pipette tip, rinsed with PBS, and treated with a dose range (0.08, 0,156, 0.312, 0.625, 1,25, and 2.5 µM) of ESE-15-one and ESE-16. FCS (0.5%) was used as a negative control and 10% FCS as a positive control. Cells were photographed then, and again after a 24 h incubation (37 °C, <5% CO_2_). Quantitation of closure of the monolayer was performed using the NIH ImageJ software (Bethesda, MD, USA) [23]. Results were expressed as a percentage of wound closed for each relevant drug concentration. Linear regression analysis was used to determine the concentration of the drug at which 50% of the wound had healed when compared to the FCS controls at 24 h. Experiments were performed in triplicate.

### 2.10. HUVEC Invasion and Migration Using xCELLigence^®^ Real-Time Cell Analysis

The cell invasion and migration (CIM) assay was conducted according to a previously described protocol [24]. Sixteen-well CIM plates (Roche Applied Science, Penzberg, Germany) with an upper and a lower chamber were assembled. Lower chambers contained serum-free medium (SFM) (negative control), SFM with 10 ng/mL basic fibroblast growth factor (bFGF) (Lonza, Basel, Switzerland) (positive control), or metastatic breast adenocarcinoma (MDA-MB-231) conditioned media. Upper chambers were prepared with 50 µL SFM per well. Plates were placed into the xCELLigence^®^ real-time cell analysis (RTCA) dual-plate instrument (Roche Applied Science, Penzberg, Germany) for 1 h before a background impedance reading was taken and the instrument was calibrated. HUVECS treated with 0.235 µM ESE-16 for 24 h were seeded into the upper chambers. The number of cells detected by electrical impedance per unit time (cell index (CI)) were recorded over 20 h on the xCELLigence^®^ RTCA DP instrument and analyzed using the RTCA software version 1.2.1 (Roche Applied Science, Penzberg, Germany) (Supplementary Methods S1.4 [25,26,27,28]).

### 2.11. Anti-Tumor and Anti-Metastatic Properties of the Novel Compounds In Ovo

The anti-tumor and anti-metastatic properties of the two estradiol analogs were assessed in vivo using chick chorioallantoic membrane (CAM) assays [29]. The green fluorescent protein (GFP)-encoding plasmid pEGFP-N1 (Clontech Laboratories, Mountain View, CA, USA) was transfected into MDA-MB-231 cells using FuGENE^®^ reagent (Roche, Indianapolis, IN, USA). Following transfection, stable GFP-positive MDA-MB-231 cell clones were isolated. Cultured MDA-MB-231-GFP cells were detached by trypsinization, washed, and suspended in serum-free DMEM. As preparation, incubation of 70 fertilized White Leghorn eggs (Société Française de Production Avicole (SFPA), St. Brieuc, France) was commenced at 38 °C with 60% relative humidity 10 days prior. On day 10 (T10), a 1 cm^2^ window was cut in the eggshell above the CAM by drilling a small hole through the shell into the air sac after the CAM was dropped. MDA-MB-231-GPF cells (1 × 10^6^) were inoculated onto the CAM of each egg. Eggs with live embryos were then randomized in 4 groups of 15 eggs. Two days later, tumors became detectable. Tumors were treated every second day (T12, T14, T16, T18) for 8 days by dropping 100 μL of either 50 μM or 25 μM of the compounds (ESE-15-one and ESE-16), 2 μM colchicine, or 0.5% DMSO (vehicle) onto the tumor. At T19, tumors resected from the upper portion of the CAM were weighed. The number of distant metastatic nodules containing MDA-MB-231-GFP-expressing cells was quantified from a 1 cm^2^ section of the lower CAM using whole mounts of fixed tissue (4% formaldehyde-PBS) using a Leica MacroFluo microscope (Leica Microsystems, Wetzlar, Germany. Data were analyzed and represented as the mean ± standard deviation (SD) of live embryos, primary tumor size, and the number of distant metastatic nodules. Data from treated samples were compared to the vehicle DMSO control using the Mann–Whitney test.

### 2.12. Statistical Analysis

Five or more representative images were captured for each sample (2 or more technical repeats) as part of qualitative microscopic evaluations. All experiments were conducted with 3 biological repeats. Quantitative data were analyzed for significance using the analysis of variance (ANOVA)-single factor model and a two-tailed Student’s *t*-test, with *p* values < 0.05 (*) taken as statistically significant. Means are depicted with bar charts, with the standard deviation shown at T-bars. Any deviations from these statistical analyses are mentioned specifically under the relevant sections.

## 3. Results

### 3.1. Temporal Analysis of the Effect of the Compounds on Cell Microtubules and Actin Filament

We analyzed the effect of ESE-15-one and ESE-16 on microtubule dynamic instability parameters using time-lapse fluorescence microscopy on GFP-EB3-transfected HeLa cells. The analysis was conducted after a 2-h exposure to ESE-15-one and ESE-16 at increasing doses using DMSO (vehicle)- and colchicine (0.05 μM)-exposed cells as negative and positive controls, respectively (Table 1 and Supplementary Videos S1–S8. The tested concentrations of ESE-15-one and ESE-16 were established based on the concentration capable of halving cell growth (IG_50_), as determined by previous studies [9,10]. All the parameters analyzed were modified by incubation with the compounds in a dose-dependent manner. These compounds, even at low doses, decreased the time that the microtubules spent growing as the time spent in pause increased. Additionally, the growth rate decreased significantly and the microtubule catastrophe frequency increased with incremental compound concentrations. The fact that distance-based catastrophe frequencies increased while time-based frequencies remained stable was attributed to the increased time spent in pause. These modifications are similar to those measured for colchicine. The lowest concentrations of ESE-16 and ESE-15-one (¼ IG_50_ i.e., 0.125 µM and 0.045 µM, respectively) showed values similar to those obtained for 0.05 µM colchicine. When compared to ESE-16, ESE-15-one had an enhanced effect on microtubule dynamics.

**Figure 2:**
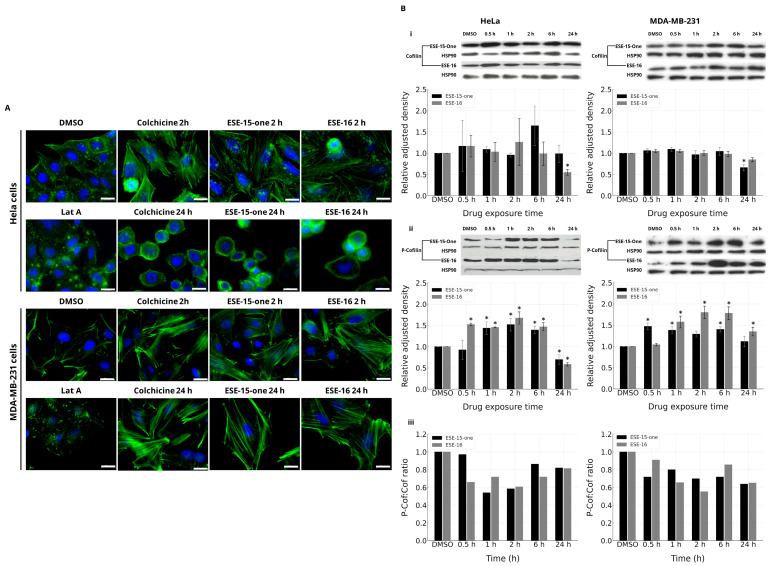
Analysis of the effect of the compounds on the actin skeleton and cofilin signaling. (**A**) Actin filaments (green) were stained with Alexa Fluor^®^ 488 Phalloidin in HeLa and MDA-MB-231 cells after exposure to the different compounds. Cells were treated with either DMSO (vehicle; <0.05% (*v*/*v*)), Latrunculin A (Lat A, 0.1 μM for 10 min), 0.1 μM colchicine, 0.186 μM ESE-15-one, or 0.5 μM ESE-16 for 2 or 24 h, as indicated. Nuclei (blue) were stained with Hoechst. Bar = 20 µm. (**B**) Western blot analysis of the sequential expression of cofilin (i) and cofilin phosphorylation (ii) in HeLa and MDA-MB-231 cells exposed to the sulfamoylated 2-ME analogs. HSP90 was used as a loading control. The results were analyzed as explained in the methods section. The P-cofilin:cofilin ratio is represented in (**B**) (iii). Bars represent the mean fold-increase as compared to DMSO of at least three repeats, the error bars show SEM, and * *p* < 0.05.

We then analyzed the effect of the compounds on the actin cytoskeleton at two time points (2- and 24-h exposures) in two different cancer cell lines (HeLa and MDA-MB-231) using fluorescent phalloidin. Latrunculin A (Lat A), a potent inhibitor of actin assembly, and the microtubule-disrupting agent colchicine, were used as controls. As expected, a short (10 min) treatment of cells with Lat A caused depolymerization of actin filaments. When compared to vehicle-exposed cells (DMSO), cells treated with colchicine for 2 h showed an increase in F-actin and thick stress fiber formation. A 24-h exposure of HeLa cells to colchicine induced rounding of the cells with a peripheral radial arrangement of the actin filaments. This was in contrast to MDA-MB-231-treated cells which remained dispersed, with prominent stress fibers visible. Interestingly, exposure of cells for 2 and 24 h to ESE-15-one and ESE-16 induced similar actin filament modifications to colchicine, with similar differences in each of the cell lines (Figure 2A).

To investigate the possible signaling behind the actin changes observed following ESE-15-one and ESE-16 exposure, cofilin expression and its phosphorylation were assessed by Western blot (Figure 2B). The cofilin-severing activity of actin filaments is inhibited by its phosphorylation, inducing prominent, stabilized stress fiber formation [30,31,32,33]. Exposure of HeLa and MDA-MB-231 cells to ESE-15-one and ESE-16 caused a significant increase in cofilin phosphorylation (between approximately a 1.2- to 2-fold increase) after 1 and 2 h of exposure, without changing the expression levels, as can be seen by the P-cofilin:cofilin ratio in Figure 2(Biii) (Table S6 in the Supplementary Results). After a 6-h treatment, the amount of phosphorylated cofilin trends back to the control level. Although the total cofilin level decreases at 24 h, probably reflecting some cell death, the ratio of phosphorylated cofilin/cofilin is similar to the control in HeLa cells.

### 3.2. The Compounds Slow Down the Migration and the Invasion of Neoplastic Cells

Tumor progression towards metastasis is a multistage process in which malignant cells spread from the primary tumor to colonize distant sites. The early steps of the metastatic cascade involve the acquisition of a motility phenotype, degradation of the extra-cellular matrix (ECM), and the establishment of productive cell–matrix interactions to invade surrounding and subsequently distant tissues.

**Figure 3:**
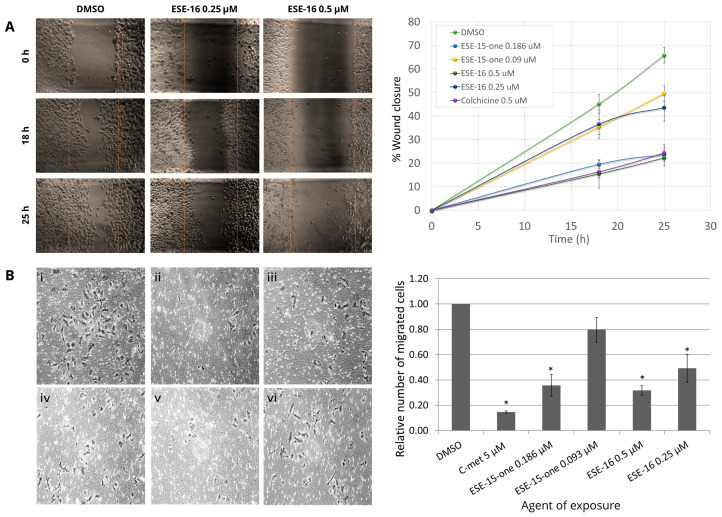
In vitro investigation of the effects of ESE-15-one and ESE-16 on cell migration and invasion. (**A**) Wound-healing assay demonstrated a dose-dependent delay in MDA-MB-231 cell migration when exposed to ESE-15-one and ESE-16. (**B**) Representative micrographs showing stained MDA-MB-231 cells which have migrated through the membrane pores towards a chemoattractant (10% FCS) when exposed to DMSO as a vehicle control (i), 5 µM selumetinib (C-met) as a positive control (ii), 0.186 µM ESE-15-one (iii), 0.09 µM ESE-15-one (iv), 0.5 µM ESE-16 (v), and 0.25 µM ESE-16 (vi). (10× magnification). Quantification of the effect of the drugs on MDA-MB-231 cell invasion was done by counting the cells which traversed the membrane. Results are reported as the relative number of cells compared to the DMSO control, as shown on the right of panel (**B**), * *p* < 0.05.

The effects of ESE-15-one and ESE-16 on the microtubule and the actin cytoskeletons could result in the inhibition of cell motility and invasion. A wound-healing assay was used to determine the effects of these compounds on the migration of cells on a 2D surface. This method mimics cell migration during wound healing in vivo. It involves creating a “wound” in a monolayer of cells and capturing images at 0, 18, and 25 h as the cells migrate to close the wound, and then comparing the degree of “wound” closure per unit time to quantify the rate of cell migration. Figure 3A shows the results of such an assay on MDA-MB-232 cells when treated with 0.25 µM and 0.5 µM ESE-16. We observed that the migration of cells treated with ESE-16 was reduced and that the wound closed in a time- and dose-dependent manner (Figure 3A). Although the effect of ESE-15-one on cell migration was less potent, similar trends were observed. These results also indicate that the compounds at their IG_50_ doses have the same order of magnitude of inhibitory activity as 0.05 µM colchicine (supporting data in Supplementary Results S2.2).

We then analyzed the effects of ESE-15-one and ESE-16 on the invasive migration of MDA-MB-231 through Matrigel^TM^ using Transwell chambers. Cells were seeded into the chamber inserts in serum-free medium, as described in the methods section. The mitogen-activated protein kinase kinase (MEK) inhibitor, selumetinib, was used as a positive control. After a 24-h incubation period, the invasive cells were counted. As shown in Figure 3B, ESE-15-one and ESE-16 exerted a dose-dependent inhibitory effect on the invasion of these cells. Exposure of MDA-MB-231 cells to 0.186 µM ESE-15-one decreased migration through the membrane pores by more than half (0.35 ± 0.09) relative to the DMSO control (taken as 1). Similar results were obtained for the 0.05 µM- and 0.25 µM-exposure of ESE-16 to the MDA-MB-231 cells (0.32 ± 0.04 and 0.49 ± 0.11, respectively). There was no significant reduction in cell migration at the lower dose of ESE-15-one in this time frame (supporting data in Supplementary Results S2.3).

### 3.3. The Effect of the Compounds on Endothelial Cell Migration

**Figure 4:**
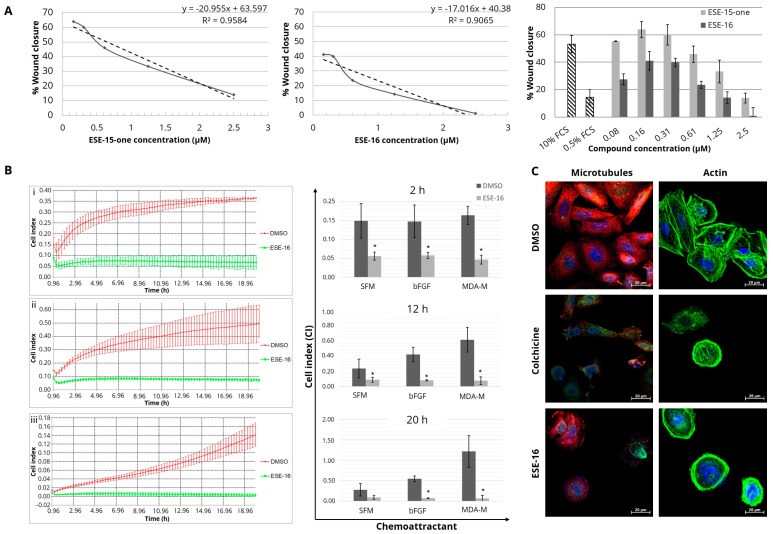
Drug-induced effects on the migration and cytoskeletal structure of human umbilical endothelial cells (HUVEC). (**A**) The effect of the drugs on HUVEC migration was assessed by healing of a wounded monolayer. Linear regression analysis was used to determine the drug concentration at which wounds had healed (50%) as compared to the FCS controls (33.8%). Results represent three biological repeats. (**B**) xCELLigence^®^ RTCA line graphs showing CI values over 20 h, with (i) SFM-, (ii) bFGF-, and (iii) MDA-MB-231-conditioned media (MDA-M) as chemoattractants, with corresponding bar charts showing CI values at 2, 12, and 20 h. CIM was highest in DMSO-exposed HUVECs moving towards MDA-MB-231-conditioned media. Overall, 0.2 µM ESE-16 inhibited CIM. Line graphs and bar charts represent the mean of three biological repeats with standard deviation indicated by error bars; * *p* value < 0.05. (**C**) Fluorescence microscopy of the HUVEC cytoskeletons: tyrosinated and detyrosinated tubulin were stained red (Cy3) and green (Alexa Fluor^®^ 488), respectively, to assess the integrity of the microtubules, whereas actin was stained green (Phalloidin-Atto 488) with blue-counterstained nuclei (Hoechst 33342) (40× magnification, 20 µm scale bar). Treated HUVECs demonstrated compromised structural integrity with abrogated microtubule dynamics and disrupted actin cytoskeletons. MDA-M = MDA-MB-231-conditioned media; SFM= serum-free medium; bFGF = basic fibroblast growth factor; CI = cell index.

Several compounds which bind to the tubulin colchicine-binding site, such as combretastatins, exhibit an anti-angiogenic effect [15]. To determine whether ESE-15-one and ESE-16 also display anti-angiogenic properties, we analyzed their effects on HUVECs. First, a cell migration experiment was performed. Linear regression analysis was used to determine the concentration of drug exposure at which the monolayer was 50% healed, compared to the 10% FCS positive control (Figure 4A). HUVEC exposure to 1.42 µM ESE-15-one inhibited wound closure by 50%, whereas 0.4 µM ESE-16 achieved the same result (supporting data in the Supplementary Results S2.4).

The effect of treatment on the ability of HUVECs to migrate towards different chemoattractants, including the MDA-MB-231 metastatic breast cancer-conditioned medium, and invade the chemoattractant-containing lower chamber, was investigated using the xCELLigence system (Figure 4B). As both compounds retarded HUVEC migration in the scratch assays, 0.2 µM ESE-16 was used as a representative in xCELLigence^®^ real-time cell analysis and fluorescent microscopy. In wells with SFM in the lower chamber, CI values, which indicate the proportion of cells that have migrated and invaded the lower chamber, were significantly reduced in HUVECs treated with ESE-16 after 2-h (0.06 ± 0.01) compared to DMSO (0.15 ± 0.05). At 12 h, the CI in ESE-16-treated HUVECs (0.09 ± 0.03) remained significantly lower than that of the DMSO-exposed cells (0.27 ± 0.11). In wells that contained bFGF as a chemoattractant in the lower chamber, the CI was significantly lower following ESE-16 treatment (0.06 ± 0.01) when compared to the DMSO control (0.15 ± 0.04). Similarly, ESE-16-treated HUVECs revealed a significant reduction in CI values (0.05 ± 0.01) towards breast cancer-conditioned media when compared to DMSO (0.16 ± 0.02) at 2 h. A similar trend prevailed at longer time points (12 and 20 h) (supporting data in the Supplementary Results S2.5).

Thereafter, using immunofluorescence, we investigated whether the compounds had the same effect on the cytoskeleton of these endothelial cells as in the neoplastic cells. The DMSO vehicle control demonstrated dynamic tyrosinated tubulin networks with structural integrity and visible nuclei in confluent monolayer cells. Actin cytoskeletons displayed prominent leading edges with lamellipodial actin filaments and normal stress fiber formation. ESE-16-treated cells revealed abrogated microtubule networks, nuclear fragmentation, and thick radial formation of the actin filaments with loss of cell polarity. As in the cancer cells, the response of HUVECs to ESE-16 was similar to colchicine-treated cells (Figure 4C).

### 3.4. Together with Cofilin Phosphorylation, P-Ezrin/Radixin/Moesin May Regulate the Actin Response to the Molecules, Which Also Activate Anti-Angiogenic, Extra-Cellular, and Adhesion Pathways

The RPPA platform was used to screen for possible pathways influenced by exposure to the investigated compounds. Results are summarized in Table 2. They indicate a trend for the expression of the various proteins to increase (upward arrows), decrease (downward arrows), or remain unchanged in the context of several signaling pathways (graphs in Supplementary Results S2.1). The most pronounced results included a sequential increase in phosphorylated ezrin/radixin/moesin, P-VEGF-R2, and TIMP2, with a decrease in P-Fyn and P-Shc in HeLa cells over 24 h. TYMP, ROCK-I/ROK beta, E-cadherin, and P-Met showed no clear change in expression or activation when compared to the negative control. The results suggest a possible variation in response between the ESE-15-one and ESE-16, as well as differences between the two cell lines investigated.

### 3.5. ESE-15-One and ESE-16 Exhibit Anti-Tumor and Anti-Metastatic Properties In Ovo

**Figure 5:**
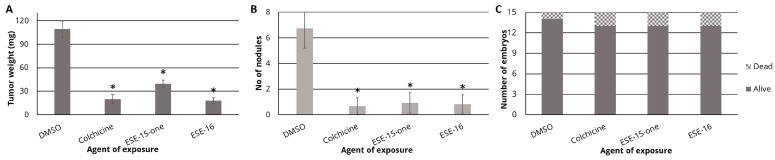
Anti-tumor and anti-metastatic effects of ESE-15-one and ESE-16. MDA-MB-231 cells were xenografted onto chick embryo chorioallantoic membranes. After treatment with either vehicle (DMSO), 2 µM colchicine, or 25 µM ESE-15-one, or ESE-16, tumors were excised and weighted. The lower CAM was also dissected and fixed, and the number of GFP-fluorescent nodules was counted on a 1 cm^2^ sample, as described in the materials and methods section. Drug treatment significantly reduced tumor mass (**A**) and the number of distant metastases (**B**), without significant toxicity to the embryos (**C**). * *p* < 0.01 represents a significant difference from the DMSO vehicle control using the Mann–Whitney test.

We used MDA-MB-231 cells expressing GFP xenografted onto chicken chorioallantoic membranes (CAM) to gain insight into the in vivo actions of the novel anti-mitotics. This method is able to determine the effect of the compounds on primary tumor mass and metastasis through the analysis of cell migration to the lower CAM (Figure S4A in the Supplementary Results). Moreover, the effect of the compounds on chick embryo viability provides insight into their toxicity (26). Drug treatment caused a significant decrease in primary tumor mass when compared to the DMSO control samples (Figure 5A). When treated with 25 μM of either compound, the tumor mass decreased from 109.46 ± 10.49 mg in the DMSO control group to 39.43 ± 5.12 mg in the ESE-15-one-exposed eggs and 17.81 ± 3.66 mg in the ESE-16-treated tumors. The number of distant metastases on the lower surface of the CAM was also significantly decreased, from 6.75 ± 1.52 in the DMSO-exposed eggs to 0.66 ± 0.65 in the colchicine-treated group, 0.92 ± 0.79 in the ESE-15-one-treated tumors and 0.83 ± 0.71 in the ESE-16-exposed chick embryos (Figure 5B). These reductions in tumor mass and the number of distant metastases are comparable to those observed with treatment with colchicine. Mortality of the treated embryos did not differ significantly from the DMSO control group (Figure 5C). By doubling the treatment dose of the compounds to 50 μM, similar effects were observed on primary tumor size and the number of distant metastases, but these higher doses resulted in increased embryo mortality (Supplementary Data in Supplementary Results S2.5).

## 4. Discussion

Microtubules and actin filaments are highly dynamic, and their dynamics are rigorously controlled by signaling pathways which are interconnected to co-ordinate mitosis, cell migration, and other cytoskeletal functions. The highly dynamic nature of microtubules is a hallmark of these structures, and is described as treadmilling and dynamic instability [34,35]. 2-ME has a pleiotropic mechanism of action which is dependent on its concentration. Kamath et al. demonstrated that low doses of 2-ME (1.2 µM) inhibited microtubule dynamics, whereas much higher concentrations were needed for microtubule depolymerization in MCF-7 breast cancer cells [36]. In this study, low-dose ranges (IG_50_, ½ IG_50_ and ¼ IG_50_) of ESE-15-one and ESE-16 were used to assess their effect on microtubule dynamic instability parameters after a 2-h exposure. We found that, at low doses, these compounds inhibited microtubule dynamics. However, a 0.5 µM concentration of ESE-16, the highest concentration assayed, induced complete microtubule depolymerization, making the distinction of the individual structures within the blebbing cells impossible. Although obtained on HeLa cells, a different line from the MCF7s studied by Kamath et al., these results strongly suggest that our compounds act similarly to 2-ME, but are more potent, since much lower doses were required to induce similar effects.

Actin filaments are necessary for cellular mobility, adhesion, and polarity. Actin stress filaments are composed of bundles of 10–30 actin filaments connected by actin-crosslinking proteins in highly dynamic interactions [37,38]. Pellegrin and Mellor (2007) noted that stress fibers in highly motile cells were sparse, thinner, and highly dynamic when compared to non-motile cells, in which they appeared thick and more stable [39]. In our experiments, we observed an increased number of thickened stress fibers in both HeLa and MDA-MB-231 cells treated with our compounds. In the DMSO control, the actin stress filaments were prominent at the leading edges of the cells, whereas treated cells did not display such polarity. The actin stress filaments of such treated cells were similar in appearance to the actin network of MDA-MB-231 cells treated with dihydromotuporamine C—a compound known to inhibit tumor invasion [40]. The thickened stress fibers were more stable and were associated with increased and enlarged focal adhesions—a response controlled by the prolonged activation of the RhoA-ROCK-signaling molecules. After 24 h, HeLa cells exposed to ESE-15-one and ESE-16 appeared rounded with a peripheral actin meshwork. This may represent the actin response to the metaphase block induced by these compounds [11,12], which is characterized by a round, ridged actomyosin cortex which facilitates cell-rounding and de-adhesion in readiness to complete mitosis [41]. MDA-MB-231 cells, however, still portrayed the prominent thickened stress fibers and a decreased cell density at this time point.

Cofilin (an actin-binding protein) is a terminal effector in the signaling cascade that is involved in actin filament treadmilling [42,43,44]. Phosphorylation of cofilin, mainly under the control of LIM motif-containing protein kinases (LimKs), LimK1 and LimK2, deactivates cofilin’s actin-severing function, resulting in the stabilization of the F-actin [45,46]. LimKs are a target for small Rho-GTPases, and are activated by phosphorylation from p21-activated kinases (PAK1, -2, and 4), ROCK 1 and ROCK 2, or myotonic dystrophy kinase-related Cdc42-binding kinase α (MRCKα) [32,45,47]. Cofilin is re-activated by dephosphorylation mediated by phosphatases from the slingshot family and chronophin [46,48].

Western blot analysis of cells exposed to a timed series of ESE-15-one and ESE-16 revealed a significant and sustained increase in P-cofilin in both cell lines. This increase was already observed after 30 min, with stable cofilin expression throughout (except at 24 h when the cells were likely undergoing advanced apoptosis). RPPA analysis did not indicate a change in ROCK 1/ROK-β. However, these data must be interpreted with caution as RPPA analysis only quantified the inactive ROCK-1/ROK-β and not the phosphorylated protein. Functional studies are therefore needed to confirm this. Furthermore, additional analysis of LimK phosphorylation would indicate whether the increased P-cofilin resulted from an activation of Limk, or from an inactivation of SSH or chronophin.

**Figure 6:**
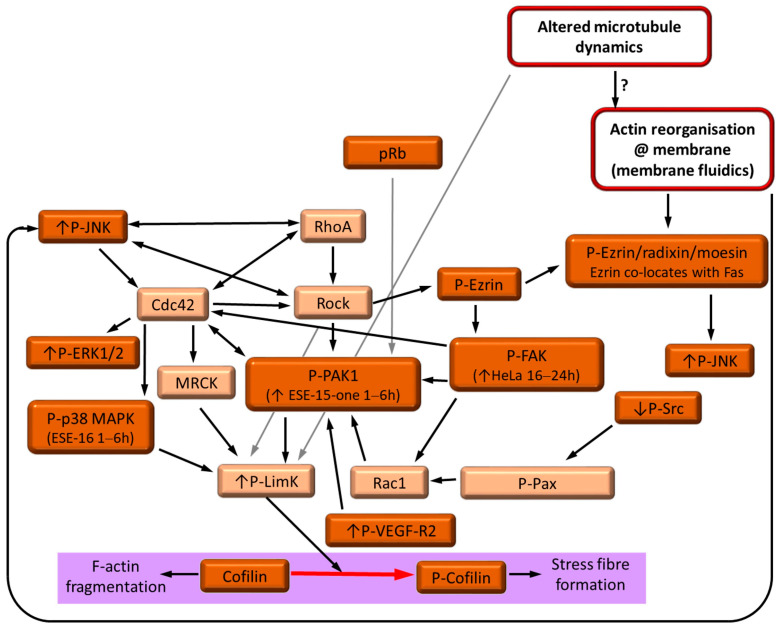
Summary of proposed pathways induced by ESE-15-one- and ESE-16 in HeLa and MDA-MB-231 cells. Proteins and events indicated in brown or red have been demonstrated with RPPA and Western blot analysis in this and previous studies [11]; pink indicates surmised pathways. Arrows indicate the direction of activation within the pathways.

When LimK is activated (either directly or indirectly), as is suggested by the increased levels of phosphorylated cofilin after ESE-15-one- and ESE-16-exposure, one would expect stabilization of the actin filaments and depolymerization of the microtubules, both of which were observed in the drug-treated cells. Additionally, P-Pak1, which may be regulated independently from RhoA, may be involved directly with actin nucleation and crosslinking proteins [49]. In the RPPA analysis, there was an indication of increased P-focal adhesion kinase (FAK) after 16 h of ESE-16 exposure. Alternatively, phosphorylation of retinoblastoma (Rb) can activate Pak, with resultant LimK-induced phosphorylation of cofilin [50]. Prominent increases of P-Rb and P-p27^kip1^ have been reported in ESE-15-one- and ESE-16-exposed HeLa and MDA-MB-231 cells [11]. The rapid temporal change in signaling may indicate a potential off-target interaction for this response, bringing into question whether the actin response to the novel compounds is solely due to microtubule dynamics changes due to alternative signaling pathways or a combination thereof (Figure 6).

Although the role of stress fibers is well described in cell-adhesion interactions, their precise role in migration remains less defined. Many highly motile cells, such as leukocytes and amoeba (*Dictyostelium discoideum*), do not demonstrate the presence of stress fibers [51,52]. Hypothetically, under certain conditions, actin stress fibers inhibit motility due to their reorganization into stable bundles [53]. Typical inhibitors of endothelial cell migration and angiogenesis, such as fumagellin, thrombospondin-1, and dihydromotuporamine C function by increasing focal adhesions and creating thick, dense stress fibers implicating the RhoA-ROCK pathway [40]. McHardy et al. (2005) reported that treatment of MDA-MB-231 cells with a meroditerpenoid increased the size and number of focal adhesions and resulted in multi-directional lamellipodia, with a decrease in cell motility and migration in cancer and vascular endothelial cells [54]. The authors reported that the molecule caused a decrease in actin stress fiber formation, but resulted in a collection of a dense meshwork of actin around the cell’s periphery.

A caveat to studying stress fibers in vitro is that they become more prominent when cells are grown on rigid surfaces. Insight may be gained by performing these investigations in a three-dimensional matrix, such as cellular spheroids, rather than in monolayer cell cultures [55,56,57]. Thus, in future mechanobiological studies, as well as in pertinent knockdown models, it would be useful to elucidate the effect of perturbed microtubule dynamics on the formation of actin stress fibers.

In the current study, a clear effect was seen on microtubule and actin morphology after ESE-15-one and ESE-16 exposure to HeLa and MDA-MB-231 cells. Signaling pathways implicated in these novel compounds’ mode of action include the GTPase pathway, in which stress fiber formation appeared to be associated with cofilin phosphorylation and increased ezrin/radixin/moesin activity. Thus, to investigate the effect of these changes on cell motility, wound-healing scratch assays were performed. Treated MDA-MB-231 monolayers demonstrated delays in wound closure, even at sub-lethal drug concentrations.

Integral to the occurrence of distant metastases is the ability of tumor cells to migrate and invade locoregional tissue and penetrate endothelial walls. Together with the capacity to migrate, cells must remodel the extra-cellular matrix to allow their passage, which may be achieved by protease digestion. The RhoA/ROCK/LimK pathway is implicated in all of the above [46]. Matrix metalloproteinases (MMPs) are a family of proteolytic enzymes which dissolve components of the ECM [58]. Tissue inhibitors of metalloproteinases (TIMPs) bind non-covalently at the active site of MMPS, inhibiting their activity [59,60]. Dysregulation of the TIMP:MMP ratio results in increased metastasis due to uncontrolled MMP activity. To investigate where ESE-15-one and ESE-16 inhibit local invasion of neoplastic cells, BD BioCoat^TM^ Growth Factor Reduced Matrigel^TM^ Invasion chambers (BD Biosciences, Franklin Lakes, NJ, USA) were used. ESE-15-one and ESE-16 significantly decreased the number of cells which invaded through the Matrigel^TM^ invasion chamber membranes. Linking in with this, TIMP2 expression increased over the 24-h drug-exposure time in both MDA-MB-231 and HeLa cells, indirectly suggesting decreased MMP activity.

Overall, there is a good correlation between the identified signaling pathways which were activated in response to the compound exposure, and the functional and morphological data we obtained. However, we cannot rule out the possibility that our compounds have targets other than tubulin, which could contribute to their therapeutic effect as well as potential toxicity and undesirable side effects. Further analysis not only on animals but also using assays commonly used in drug development will enable us to clarify these points.

MDAs have demonstrated good anti-neovascularization and anti-angiogenic properties. Not only do they inhibit cell division and migration by altering cytoskeletal and focal adhesion dynamics, but they also interfere with the shuttling of transcription factors from the cytosol to the nucleus for transcriptional activation of relevant pathways. Ample literature has been published on the anti-angiogenesis effects of the parent compound 2-ME [61,62,63]. Mechanisms in which 2-ME and its analogs inhibit angiogenesis in vitro and in vivo involve dysregulation of HIF-1α signaling [62,64]. By inhibiting microtubule dynamics, the actin skeleton is reorganized, resulting in capillary permeability via the Rho-ROCK pathway [65]. During angiogenesis, activated endothelial cells migrate towards pro-angiogenic factors and invade the extra-cellular matrix [66,67]. To investigate whether ESE-15-one and ESE-16 may potentially inhibit migration, scratch assays were done in vitro. Healing of the wound created in a confluent HUVEC monolayer was used to indicate the potential inhibitory effect of the novel compounds on endothelial cell migration. Higher doses of ESE-15-one (1.42 µM) were required to inhibit wound closure by 50%, while the same effect was obtained with only 0.4 µM of ESE-16. RTCA of HUVEC invasion and migration after a 24-h exposure to 0.2 µM ESE-16 demonstrated suppression of the CI in a sustained manner. In addition, double-fluorescence staining of ESE-16-treated HUVECs showed decreased cell density, cell rounding, nuclear fragmentation, and microtubule abrogation. Actin staining revealed disruptions in cell polarity with a loss of leading edges, a thick radial arrangement, and increased stress fibers. These changes in the actin cytoskeleton affect not only cell morphology but cytokinesis and cell locomotion as well [68].

The mechanism behind the anti-angiogenic properties of a drug relies on the inhibition of endothelial cell proliferation, as well as on preventing the migration and formation of new vessels [69]. Angiogenesis is largely controlled by vascular endothelial growth factor (VEGF) and its binding to the membrane tyrosine receptor kinases VEGF-R1 and –R2. Downstream signaling may involve MAPK, phosphoinositol-3-kinase (PI3K), Pak, and the Rho-GTPases [70,71,72]. Angiogenesis is initiated by the binding of VEGF secreted by hypoxic tumor cells under the control of hypoxic-inducible factor 1 (HIF-1α) to VEGF-R2 on endothelial cells [73]. However, VEGFR-2 is also expressed on certain other cells, including breast cancer cells [74]. The literature links this receptor to many of the signaling pathways already discussed in relation to actin and microtubule dynamics (depending on the phosphorylation sites). Upon activation, the receptor recruits VEGF-receptor-associated proteins (VRAPs), which are normally associated with Src and phosphorylated PI3K [75]. Src is a non-receptor tyrosine kinase which mediates actin organization and thus regulates the associated actions. The Rho-GTPase pathway may be induced by Y1175, and pY1175 phosphorylation of VEGF-R2, as this recruits the SH2 domain-containing adaptor protein B (SHB), which activates focal adhesion kinase (FAK), which in turn activates Rac1 and initiates actin stress fiber formation. Lastly, P-VEGFR-2 (Y1214) recruits CDK, which activates Src family kinase Fyn and results in Pak-2 activation [76]. This then activates Cdc42 and mitogen-activated kinase p38, which is responsible for actin stress fiber formation [77].

To investigate whether the novel compounds retained their anti-cancer properties in vivo at acceptable toxicity levels, CAM in ovo models were used [29]. Treatment of tumors induced by inoculating MDA-MB-231 cells with ESE-15-one and ESE-16 resulted in a significant reduction in tumor mass, as well as significantly fewer metastases on the lower CAM, while being well tolerated.

Our data do not yet provide insight into whether the anti-angiogenic effect or the cytotoxic effect will dominate in in vitro tumors, or whether it will be a combination of the two. Further understanding of the pharmacokinetics of these compounds, combined with in vivo analysis using intravital microscopy, could provide some answers.

## 5. Conclusions

ESE-15-one and ESE-16 are in silico-designed 2-ME analogs designed to increase potency and oral bioavailability, circumvent hepatic steroid metabolism, and selectively bind to CAIX in the tumor micromilieu. Data indicate that ESE-15-one and ESE-16 inhibit cell motility, migration, and invasion via microtubule dynamics dysregulation. Conformational changes of the F-actin, controlled by cofilin phosphorylation, were induced potentially via the ezrin/radixin/moesin-small GTPase signaling pathways, which resulted in thickened, non-dynamic stress fiber formation. The precise mechanisms of these signaling pathways need further investigation. Anti-invasion may be ascribed to the reduction in cellular migration, as well as moderating signaling pathways relating to the extra-cellular milieu. This was substantiated by increased TIMP2 expression, which tied in with previous studies indicating decreased CAIX activity. Cytoskeletal abrogation and decreased migration in HUVECs warrant further investigation to confirm the anti-angiogenic properties of the compounds. CAM studies link the in vitro investigations to a living model, demonstrating anti-tumor and anti-metastatic properties with an acceptable toxicity profile. Future research will focus on murine models to establish the value of these compounds as monotherapy or together with radiation as combination therapy.

## Figures and Tables

**Table 1:** T Alteration of microtubule dynamic instability parameters by increasing doses of ESE-15-one and ESE-16. Values are averages of two repeats (tracking of a minimum of eight microtubule tips per cell, with a minimum of six cells per condition) ± standard error of the mean (SEM). * *p* < 0.05; ** *p* < 0.01; *** *p* < 0.001, significantly different from control values (DMSO) using the Student’s *t* test. TD = total depolymerization of the microtubule network.

Parameters	DMSO (0.05% *v*/*v*)	Colchicine 0.05 µM	ESE-16 0.125 μM (¼ IG_50_)	ESE-16 0.25 μM (½ IG_50_)	ESE-16 0.5 μM (IG_50_)	ESE-15-One 0.045 μM (¼ IG_50_)	ESE-15-One 0.09 μM (½ IG_50_)	ESE-15-One 0.186 μM (IG_50_)
% Time spent growing	80.44	58.4 *	62.08 *	29.18 ***	TD	58.92 **	37.51 ***	25.46 ***
% Time spent in pause	19.56	41.60 *	37.32 *	70.82 ***	TD	41.08 **	62.03 ***	74.54 ***
Growth rate (µm/min ± SEM)	16.18 ± 0.42	14.61 ± 0.36 *	14.04 ± 0.51 *	9.8 ± 0.64 ***	TD	13.74 ± 0.52 *	11.91 ± 0.3 ***	10.298 ± 0.51 **
Catastrophe frequency (µm^−1^ ± SEM)	0.12 ± 0.016	0.29 ± 0.016 ***	0.21 ± 0.01 **	0.89 ± 0.06 ***	TD	0.20 ± 0.02 *	0.28 ± 0.03 *	0.89 ± 0.13 **
Catastrophe frequency (min^−1^ ± SEM)	1.56 ± 0.21	2.38 ± 0.24	1.65 ± 0.21	2.52 ± 0.3 *	TD	1.53 ± 0.11	1.2 ± 0.13	1.85 ± 0.14

**Table 2:** RPPA results from the temporal exposure of ESE-15-one and ESE-16 to HeLa and MDA-MB-231 cells. Indicated is the antibody tested, the pathway involved, and the visual trend observed in expression. Individual graphs of each array are included in the Supplementary Data (↑ minimal increase; ↑↑ increase; ↑↑↑ pronounced increased; ↓ decrease; - no change from control).

Antibody Name	Pathway(s)	HeLa		MDA-MB-231	
		**ESE-15-One**	**ESE-16**	**ESE-15-One**	**ESE-16**
MAPK/Erk signaling, cytoskeleton					
P-Ezrin (T567)/radixin (T564)/moesin (T558)	PI3K pathway, MAPK/Erk signaling, cytoskeleton	↑↑↑ over 24 h	↑↑↑ over 24 h	↑↑ over 24 h	↑↑ over 24 h
P-Shc (Y239/240)	Tyrosine kinase & MAPK/Erk signaling	↓ 6–24 h	↓ 6–24 h	-	-
P-p38 MAPK (T180/Y182)	MAPK/Erk signaling	↑ 1–2 h	↑ 1–2 h	-	↑ 1–2 h
Angiogenesis, matrix metalloproteases, extra-cellular matrix					
TIMP2	Angiogenesis, matrix metalloproteases, extra-cellular matrix	↑↑ over 24 h	↑↑ over 24 h	↑↑ over 24 h	↑↑ over 24 h
P-VEGF R2 (Y1214)	Angiogenesis	↑↑ over 24 h	↑↑ over 24 h	↑↑ over 24 h	↑↑ over24 h
Tyrosine kinase signaling, adhesion					
P-Fyn (Y528)/Src (Y530)	Tyrosine kinase signaling, SRC family	↓ 6–24 h	↓ over 12–24 h	-	-
P-FAK (Y861)	Tyrosine kinase signaling, Adhesion	↑ 1–2 h	↑ 1–24 h	↑ 1–2 h	↑ 1–24 h
Proteins apparently not affected by compound exposure					
	E-cadherin, ROCK-I/ROK beta, P-Met, TYMP				

## Data Availability

All data generated or analysed during this study are included in this published article [and its Supplementary Information File].

## Supplementary Materials

The following supporting information can be downloaded at: https://www.mdpi.com/article/10.3390/cancers16172941/s1: Supplementary methods and results. The supplementary methods consist of: S1.2 Buffer compositions; S1.2 Cytoskeletal morphology: fluorescent examination of tyrosinated- and detyrosinated tubulin and actin; Table S1 Antibodies used in RPPA analysis; and S1.4 Cell invasion and migration using xCELLigence^®^ real-time cell analysis. The supplementary results consist of the following: Figure S1. RPPA results from 0.186 µM ESE-15-one- and 0.5 µM ESE-16-exposure of HeLa and MDA-MB-231 cells at various time points over 24 h; Figure S2. Wound healing micrographs of MDA-MB-231 cells exposed to ESE-15-one and ESE-16; Table S2. Data obtained from wound healing experiments; Table S3 Statistical analysis of migrated cells relative to DMSO; Table S4. Human umbilical endothelial cell-migration assay statistics; Figure S3. Bar chart representing % wound closed of HUVEC cells at various drug concentrations, as opposed to 0.5 and 10% FCS; Table S4: HUVEC invasion and migration at 2, 6, 12 and 20-h; Figure S4. Anti-tumor and anti-metastatic properties of ESE-15-one and ESE-16 in ovo; Table S5. Effect of ESE-15-one and ESE-16 on tumor mass, number of distant metastases and assessment of toxicity using chick chorioallantoic membrane assays; and Table S.6. Ratio of Cof:P-Cof expression in HeLa and MDA-MB-231 cells over the 24-hour-drug exposures. Representative videos of the microtubule dynamics in response to various drug concentrations have been included: Video S1_DMSO MT dynamics; Video S2_ Colchicine 0.05 µM MT dynamics; Video S3_ESE-15-one_0.186 µM MT dynamics; Video S4_ESE-15-one_0.09µM MT dynamics; Video S5_ESE-15-one_0.045 µM MT dynamics; Video S6_ESE-16_0.5 µM MT dynamics; Video S7_ESE-16_0.25 µM MT dynamics; and Video S8_ESE-16_0.125 µM MT dynamics.
