# Supplementary material for: Sulfamoylated Estradiol Analogs Targeting the Actin and Microtubule Cytoskeletons Demonstrate Anti-Cancer Properties In Vitro and In Ovo
Source: Cancers (Basel). 2024 Aug 23;16(17):2941. doi: 10.3390/cancers16172941 (PMC11394244; doi:10.3390/cancers16172941)
Supplement: Supplementary file 1 [file cancers-16-02941-s001.zip › Mercier et al Supplementary material 2024_r delinked.pdf]

Supplementary methods and results

Sulfamoylated estradiol analogues targeting the actin and microtubule cytoskeletons demonstrate anti-cancer properties *in vitro* and *in ovo*

Mercier AE*, Joubert AM, Prudent R, Viallet J, Desroches-Castan A, De Koning L, Mabeta P, Helena J, Pepper MS & Lafanechère L

*** Correspondence:** Anne Mercier: joji.mercier@up.ac.za

Cancers 2024

S1. Supplementary methods

S1.2 Buffer compositions

Laemmli buffer: 50 mM Tris (Ph 6.8), 2% sodium dodecyl sulphate (SDS) 5% glycerol, 2.5 mM ethylenediaminetetraacetic acid (EDTA), 2.5 mM ethylene glycol tetraacetic acid (EGTA) and double distilled water (chemicals from Thermo Fisher Scientific, Waltham, MA, USA) with 50 µl Halt phosphatase cocktail (Thermo Fisher Scientific, Waltham, MA, USA), 1 tablet/5 mL protease inhibitor cocktail (complete MINI EDTA-free from Sigma-Aldrich (St Louis, MO, USA), 4 mM sodium orthovanadate and 20 mM sodium fluoride (Merck Millipore Corp., Darmstadt, Germany).

RIPA cell lysis buffer: 150 mM NaCl, 10 mM Tris-HCl, pH 7.4, 0.1% SDS, 0.5% sodium deoxycholate, 1mM EDTA, 1mM EGTA, protease inhibitor cocktail 2 (Sigma-Aldrich) and phosphatase cocktail (Sigma-Aldrich, St. Louis, MO, USA)

Western blot 1X migration buffer: 25 mM Tris, 190 mM glycine, 0.1% SDS, pH8.3

Western blot transfer buffer: 25 mM Tris, 190 mM glycine, 20% ethanol, pH 8.3

S1.2 Cytoskeletal morphology: fluorescent examination of tyrosinated- and detyrosinated tubulin and actin

Cy3 (excitation wavelength (λ_{ex}): 550 nm; emission wavelength (λ_{em}): 570 nm), Alexa Fluor® 488 (λ_{ex}: 490 nm; λ_{em}: 525 nm), Phalloidin-Atto 488 (λ_{ex}: 488 nm; λ_{em}: 590 nm) and Hoechst 33342 (λ_{ex}: 350 nm; λ_{em}: 461 nm) fluorescence were viewed with a Zeiss laser scanning microscope (LSM) 880 with Airyscan (Jena, Germany) [Laboratory for Microscopy and Microanalysis, University of Pretoria, Pretoria, SA].

S1.3 Signal transduction: Reverse phase protein array platform

Table S1. Antibodies used in RPPA analysis. Sp = species in which antibody was raised (R=rabbit, M=mouse); suppliers: CST= Cell Signaling Technology Corp.; Upstate (Merck Millipore Corp.); BD = BD Biosciences; Sigma = Sigma-Aldrich; Ref = reference catalogue number.

Name	Sp	Supplier	Ref	Dilution	Pathways
**Tyrosine kinase signaling, mitogen-activated protein kinase (MAPK)/ extracellular-signal-regulated kinases (Erk) signaling, cytoskeleton**					
Phospho-ezrin (Thr567)/ radixin (Thr564)/ moesin (Thr558)	R	CST	'3141'	1/10000	PI3K pathway, MAPK/Erk signaling, Cytoskeleton
Rho-associated, coiled-coil-containing protein kinase 1 (ROCK-I)/ROK beta)	R	CST	'4035 (C8F7)'	1/500	Cytoskeleton, Adhesion
Phospho-p38 mitogen-activated protein kinase (MAPK) (Thr180/Tyr182)	M	BD	'612288'	1/500	MAPK/Erk signaling
E-cadherin	M	BD	'610181'	1/250	Cytoskeleton, Adhesion
Phospho-sarcoma (Src) homology 2 domain containing) Shc (Tyr239/240)	R	CST	'2434'	1/1000	Tyrosine kinase signaling, MAPK/Erk signaling
**Angiogenesis/ matrix metalloproteases/ extracellular matrix**					
Thymidine phosphorylase (TYMP)	R	Sigma	'HPA001072'	1/250	Angiogenesis, Apoptosis
Tissue inhibitors of metalloproteinases 2 (TIMP2)	R	CST	'5738'	1/1000	Angiogenesis, matrix metalloproteases, extracellular matrix
Phospho-vascular endothelial growth factor receptor2 (VEGFR2) (Tyr1214)	R	Novus	'NB100-92662'	1/1000	Angiogenesis
**Tyrosine kinase signaling/adhesion**					
Phospho-mesenchymal epithelial transition factor (Met) (Tyr1234)	R	GeneTex	'GTX50262'	1/500	Tyrosine kinase signaling
Phospho-Fyn (Tyr528) / Src (Tyr530)	M	BD	'612668'	1/1000	Tyrosine kinase signaling, SRC family
Phospho-focal adhesion kinase (FAK) (Tyr861)	R	Epitomics	'2153-1'	1/500	Tyrosine kinase signaling, adhesion

S1.4 Cell invasion and migration using xCELLigence® real-time cell analysis

The degradation of the extracellular matrix at the onset of metastasis is characterized by endothelial cell migration into the surrounding stroma, stimulated by angiogenic chemokines. Trans-well assays enable quantification of endothelial cell migration in response to specific chemokines and growth factors [25]. SFM served as a negative chemotactic control [26]. bFGF, a potent angiogenic factor which is overexpressed in breast cancer patients and is linked to chemoresistance, was employed as a chemoattractant positive control [27,28]. The cell invasion and migration (CIM) assay used in this study has been described by Mabeta *et al.*[24].

HUVECs were seeded into 25 cm^2 flasks (750 000 cells/5 ml). At 24-h following drug exposure, trypsinized cells were resuspended in supplemented EBM™ without FBS (serum-free medium (SFM)). CIM plates (Roche Applied Science, Penzberg, Germany) were assembled. Lower chambers contained 160 µl SFM (negative control), SFM with 10 ng/ml bFGF (Lonza, Basel, Switzerland) (positive control), or -breast adenocarcinoma (MDA-MB-231) conditioned media. Upper chambers were prepared with 50 µl SFM. Plates were placed into the xCELLigence® real-time cell analysis (RTCA) dual plate (DP) instrument (Roche Applied Science, Penzberg, Germany) for 1-h to allow the membrane (upper chamber) to reach equilibrium with the media (lower chamber). A background impedance reading was taken and the instrument was calibrated. HUVECs were seeded into upper chambers (6 000 cells/100

µl) and incubated at RT for 30 min. Plates were then loaded onto the xCELLigence® RTCA DP instrument.

The number of cells detected by electrical impedance per unit time (cell index (CI)) was recorded over 20-h and analyzed using the RTCA software version 1.2.1 (Roche Applied Science, Penzberg, Germany).

S2. Supplementary results

S2.1. RPPA analysis

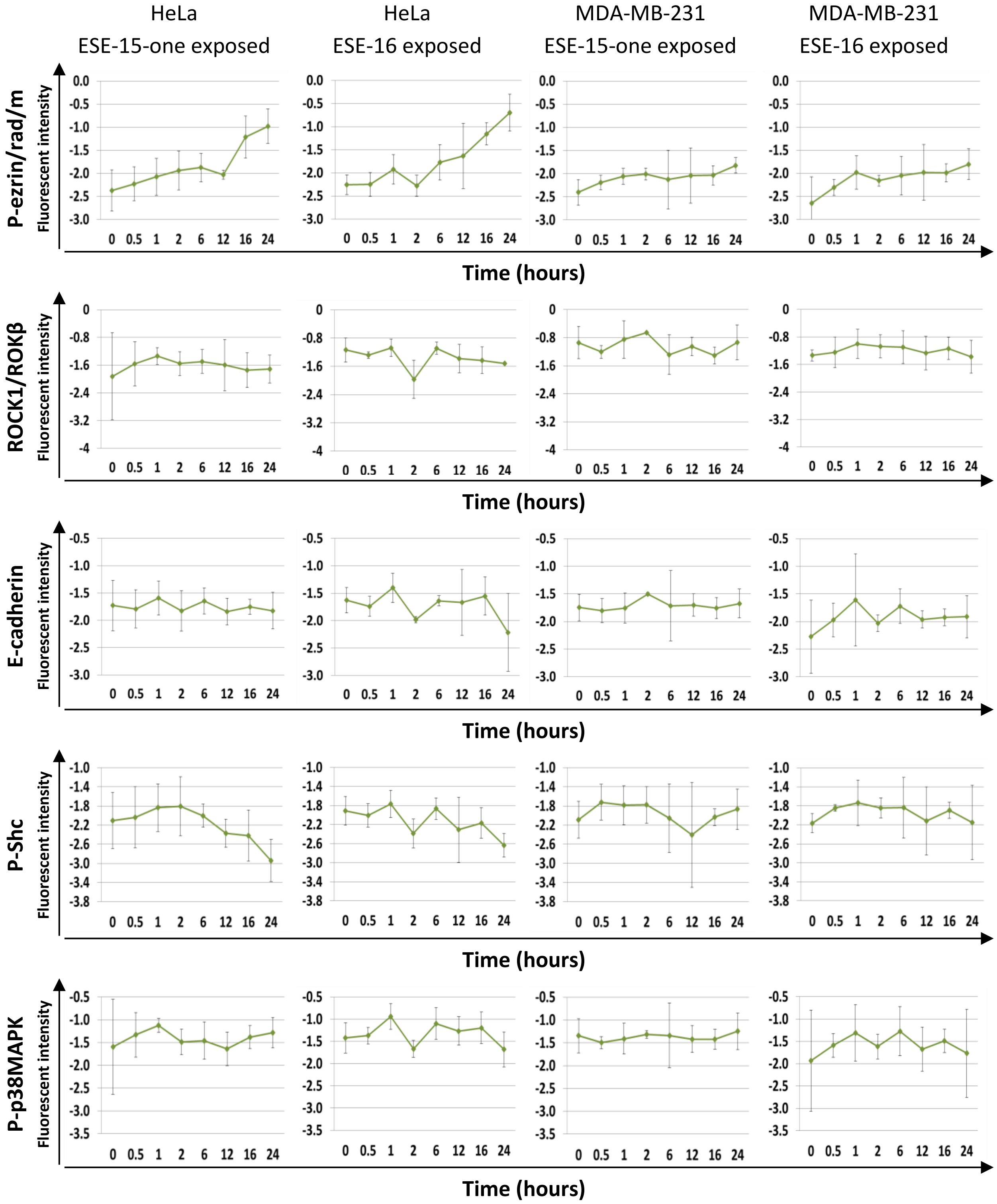

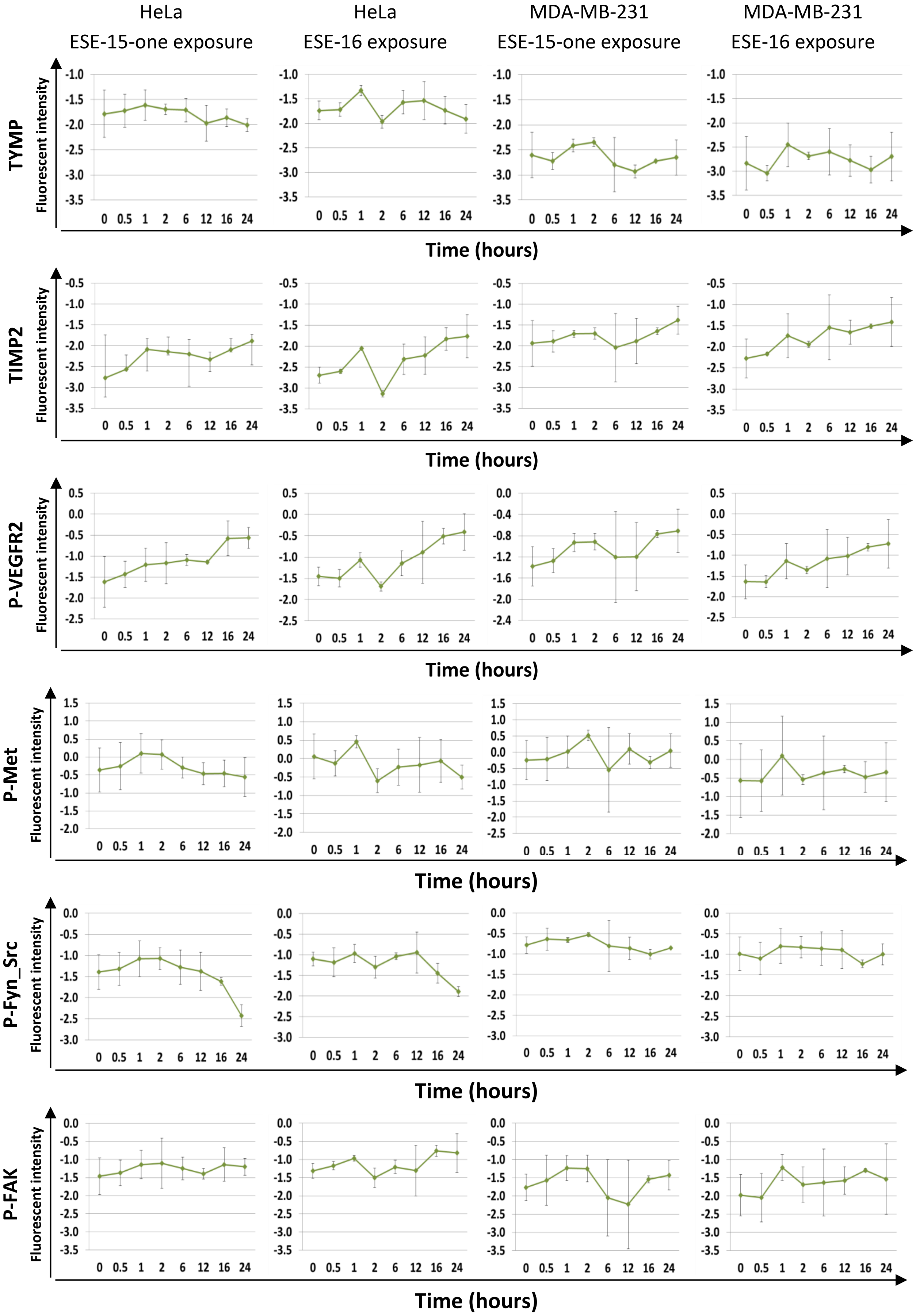

Figure S1. RPPA results from 0.186 µM ESE-15-one- and 0.5 µM ESE-16-exposure of HeLa and MDA-MB-231 cells at various time points over 24 hours.

S2.2 Wound healing assays

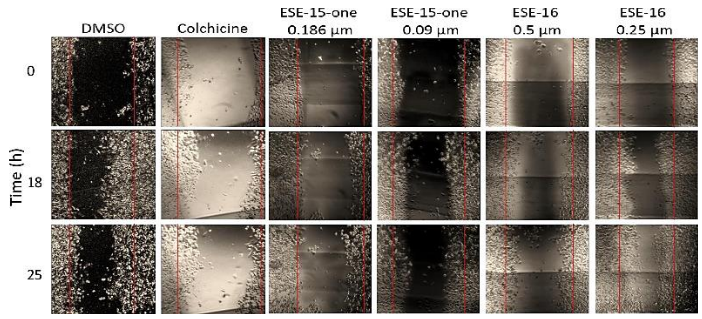

Figure S2. Wound healing micrographs of MDA-MB-231 cells exposed to ESE-15-one and ESE-16, as well as the various controls. DMSO was used as a negative vehicle control to which the drug-exposed conditions were compared, at various time intervals. (4x magnification)

Table S2. Data obtained from wound healing experiments. Confluent MDA-MB-231 cells were wounded, after which they were exposed to the IG_{50} concentration of the novel compounds, as well as half thereof. % Wound healing was calculated as the mean of 3 biological repeats with the standard error of the mean (SEM), **P* value <0.05.

	% Wound Closure	DMSO	Colchicine 0.5 µM	ESE-15-one 0.186 µM	ESE-15-one 0.09 µM	ESE-16 0.5 µM	ESE-16 0.25 µM
Hours							
0	Average	0	0	0	0	0	0
	SEM	0	0	0	0	0	0
18	Average	45.2	16.33	19.5	35.40	15.45	36.52
	SEM	3.94	2.60	1.67	3.25	6.01	6.08
	P Value		0.013*	0.014*	0.114	0.028*	0.233
25	Average	65.78	24.24	23.7	49.63	22.18	43.57
	SEM	3.24	3.58	3.02	3.26	3.32	5.78
	P Value		0.007*	0.005*	0.038*	0.006*	0.042*

S2.3 Transwell assays invasion assays

Table S3 Statistical analysis of migrated cells relative to DMSO.

	DMSO	C-met 5 µM	ESE-15-one 0.186 µM	ESE-15-one 0.093 µM	ESE-16 0.5 µM	ESE-16 0.25 µM
Relative amount of migrated cells (mean)	1,00	0,15	0,36	0,80	0,32	0,49
Stddev		0,01	0,12	0,14	0,05	0,16
SEM		0,01	0,09	0,10	0,04	0,11
P value		0,00	0,02	0,17	0,00	0,04

S2.4 Anti-angiogenesis investigation using HUVEC scratch assays

Table S4. Human umbilical endothelial cell-migration assay. Statistics represented the average wound closure (3 repeats) over 24 h, with standard deviations (Std Dev) and coefficient of variations (CV).

			Average	STD Dev	CV
ESE-15-one		10% FCS	0.00	0.00	0.12
		0.5% FCS	14.50	5.67	0.39
	0.08	0.08	55.23	0.20	0.00
	0.16	0.16	63.81	5.92	0.09
	0.31	0.31	59.92	7.45	0.12
	0.61	0.61	45.87	5.96	0.13
	1.25	1.25	33.28	8.32	0.25
	2.5	2.5	13.90	3.54	0.25
ESE-16		10% FCS	53.19	6.29	0.12
		0.5% FCS	0.00	0.00	0.39
	0.08	0.08	27.52	4.03	0.15
	0.16	0.16	41.09	6.72	0.16
	0.31	0.31	39.76	3.13	0.08
	0.61	0.61	23.59	2.64	0.11
	1.25	1.25	14.22	4.34	0.31
	2.5	2.5	1.05	6.01	5.71

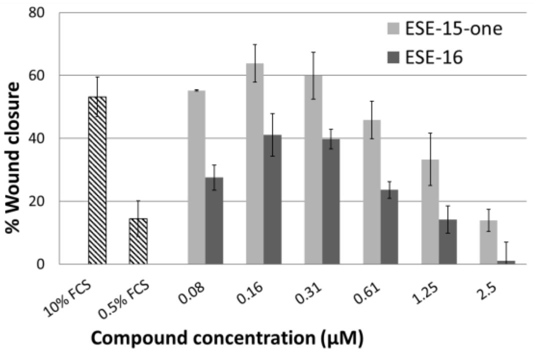

Figure S3. Bar chart representing % wound closed of HUVEC cells at various drug concentrations, as opposed to 0.5 and 10% fetal calf serum (FCS).

S2.5 xCELLigence® real-time cell analysis

Table S4: HUVEC invasion and migration at 2, 6, 12 and 20-h. Mean (CI) and standard deviation (SD CI) represent CIM in 2 biological repeats (each with n=3). Statistically significant differences (*P* value < 0.05) are indicated by *.

Time		2 h		6 h		12 h		20 h	
Agent of exposure		DMSO	ESE-16	DMSO	ESE-16	DMSO	ESE-16	DMSO	ESE-16
Chemo-attractant									
SFM	Mean (CI	0.15	0.06	0.25	0.09	0.27	0.09	0.28	0.09
	SD (CI)	0.05	0.01	0.07	0.02	0.11	0.03	0.15	0.05
	P value cf DMSO		2.26E-02*		2.09E-02*		4.73E-02*		1.32E-01
	P value cf 2 h			1.20E-01	1.50E-01	1.42E-01	1.82E-01	2.29E-01	2.88E-01
	P value cf 6 h					7.36E-01	9.11E-01	7.59E-01	8.31E-01
	P value cf 12 h							9.70E-01	9.02E-01
bFGF	Mean (CI	0.15	0.06	0.27	0.08	0.32	0.08	0.37	0.07
	SD (CI)	0.04	0.01	0.11	0.01	0.17	0.01	0.24	0.01
	P value cf DMSO		2.40E-02*		3.82E-02*		7.12E-01		1.06E-01
	P value cf 2 h			1.39E-01	1.45E-02*	1.62E-01	3.93E-02*	2.02E-01	9.25E-02
	P value cf 6 h					6.56E-01	6.02E-01	5.57E-01	1.83E-01
	P value cf 12 h							8.02E-01	3.74E-01
MDA-MB-231 medium	Mean (CI	0.16	0.05	0.34	0.07	0.61	0.07	1.21	0.07
	SD (CI)	0.02	0.01	0.07	0.03	0.156	0.05	0.39	0.07
	P value cf DMSO		1.64E-03*		1.45E-02*		5.50E-03*		7.48E-03*
	P value cf 2 h			1.67E-02*	2.66E-01	9.17E-03*	4.21E-01	9.61E-03*	6.35E-01
	P value cf 6 h					5.89E-01	9.55E-01	1.89E-02*	9.39E-01
	P value cf 12 h							6.86E-02	9.15E-01

CIM to SFM (negative control)

CI values were significantly lower in the ESE-16-treated HUVECs (0.06 ± 0.01) after 2-h (DMSO 0.15 ± 0.05). A significant decrease in CI value was noted in the treated cells at 6-h. At 12-h, CIM in ESE-16-treated HUVECs (0.09 ± 0.03) remained significantly lower than in DMSO-exposed cells (0.27 ± 0.11), although the migration rate was not significant in the control group at the later time points, thus not rendering any difference at 20 h.

CIM to SFM supplemented with bFGF (positive control)

After 2-h, CIM was significantly lower in the ESE-16 treated cells (0.06 ± 0.01) than in DMSO (0.15 ± 0.04). CI values were significantly reduced in the ESE-16- (0.08 ± 0.01) treatment (0.09 ± 0.01) at 6-h (DMSO 0.27 ± 0.11).

CIM to MDA-MB-231 conditioned media

At 2-h, the ESE-16-treated cells (0.05 ± 0.01) revealed significant reductions in CIM towards breast cancer conditioned media when compared to DMSO (0.16 ± 0.02). A similar trend prevailed at 6-, 12- and 20h. CIM was significantly higher in the DMSO control at 12- and 20-h than at 2 and 6 h.

S2.6 In ovo experiments

When treated with the 50 µM dose, the control tumor weighed 159.9 ± 26.5 mg, the colchicine-, ESE-15-one- and ESE-16-treated tumors weighed 44.8 ± 20.9 mg, 74.3 ± 13.77 mg, and 59.3 ± 4.85 mg, respectively (Figure S4 and Table S5). Additionally, treated specimens had reduced numbers of distant nodules on the lower surface of the CAM. The DMSO control had 12.2 ± 2.14 nodules, colchicine-exposed tumors 1.8 ± 21.17, ESE-15-one treated specimens 2.2 ± 1.72 and ESE-16 treated embryos had 1.5 ± 1.05 distant metastases. This experimental condition did, however, result in high mortality rates of the embryos when treated with 50 µM ESE-15-one and ESE-16, with 7 and 9 embryos dying respectively. The DMSO and colchicine-exposed embryos only reported a demise of 2.

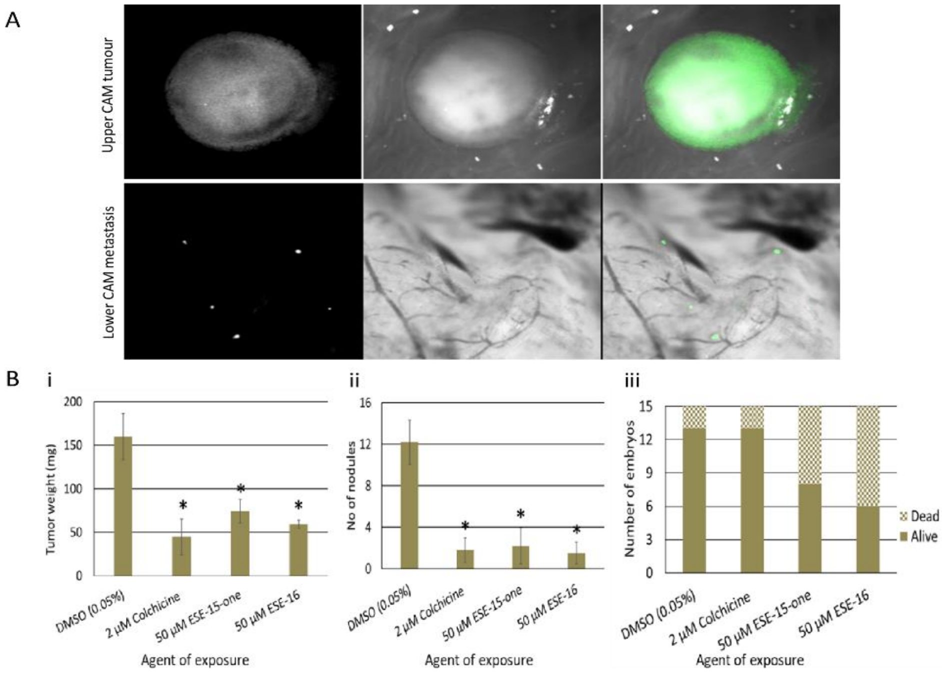

Figure S4. Anti-tumor and anti-metastatic properties of ESE-15-one and ESE-16 *in ovo.* MDA-MB-231 cells xenografted onto CAMs were treated with ESE-15-one and ESE-16 (50 µM) while DMSO was used as the vehicle control, and 2 µM colchicine as a positive control. A 1 cm^2 sample of the lower CAM was viewed with fluorescence microscopy to quantify the number of distant nodules. (A). Representative images of a tumor on the upper CAM, and the metastasis on the lower CAM. (B). Drug treatment at 50 µM reduced the tumor size (Bi) and the number of distant metastases (Bii) significantly but demonstrated some toxicity to the embryos (iii). **P* < 0.01 represents a significant difference from the DMSO vehicle control using the Mann-Whitney test.

Table S5. Effect of ESE-15-one and ESE-16 on tumor mass, number of distant metastases and assessment of toxicity using chick chorioallantoic membrane assays. Data represents the mean ± standard deviation of 15 embryos within each group. Experiment 1 comprised of treatment concentrations of 50 µM, whereas experiment 2 halved the drug-exposure dose to 25 µM. The Mann-Whitney test was used to analyze statistical significance of the reduction of tumor size and number of distant metastases.

Drug of Exposure	Tumour weight (mg)	Std dev	*P* (DMSO)	No of nodules	Std dev	*P* (DMSO)	Alive	Dead
**Experiment 1: 50 µM of the compounds**								
DMSO (0,05%)	159.9	26.5		12.2	2.14		13	2
2 µM Colchicine	44.8	20.9	3.74E-06	1.8	1.17	1,17 E-04	13	2
50 µM ESE-15-one	74.3	13.77	8.70E-05	2.2	1.72	3.39E-04	8	7
50 µM ESE-16	59.3	4.85	9.32E-05	1.5	1.05	1.7E-05	6	9
**Experiment 2: 25 µM of the compounds**								
DMSO 0,02%	109.46	10.49		6.75	1.54		14	1
2 µMColchicine	19.82	6.04	1.15E-13	0.66	0.65	3.5E-08	13	2
25 µM ESE-15-one	39.43	5.12	1.41E-11	0.92	0.79	6.3E-08	13	2
25 µM ESE-16	17.81	3.66	2.48E-12	0.83	0.71	4.7E-08	13	2

S2.7 Ratio of Cof:P-Cof expression

Table S.6. Ratio of Cof:P-Cof expression in HeLa and MDA-MB-231 cells over the 24-hour-drug exposures.

**Ratio of Cof expression:P-Cof phsophorylation**						
Time of exposure	0 h	30 min	1 h	2 h	6 h	24 h
HeLa cells						
ESE-15-one						
Ave ratio ± SD	1:1	1:1.03 ± 0.62	1:1.85 ± 0.4	1:1.71 ± 0.41	1:1.16 ± 0.06	1:1.22 ± 0.26
P-value		9.33E-01	**2.14E-02**	**3.81E-02**	**1.90E-02**	5.82E-01
ESE-16						
Ave ratio ± SD	1:1	1:1.52 ± 0.06	1:1.39 ± 0.23	1:1.65	1:1.39 ± 0.21	1:1.23 ± 0.55
P-value		**1.17E-04**	**4.24E-02**	**2.74E-02**	**3.43E-02**	5.07E-01
MDA-MB-231 cells						
ESE-15-one						
Ave ratio ± SD	1:1	1:1.39 ± 0.17	1:1.25 ± 0.11	1:1.43 ± 0.44	1:1.39 ± 0.31	1:1.57 ± 0.1
P-value		**2.41E-04**	**7.20E-04**	**3.78E-02**	**8.46E-03**	**2.28E-06**
ESE-16						
Ave ratio ± SD	1:1	1:0.99 ± 0.12	1:1.53 ± 0.4	1:1.81 ± 0.48	1:1.7 ± 0.27	1:1.54 ± 0.18
P-value		9.20E-01	**9.89E-03**	**2.79E-03**	**1.14E-04**	**3.82E-05**

S3. References

Under references in the manuscript.
